# The Role of the Indigenous Patient Navigator: A Scoping Review

**DOI:** 10.1177/08445621211066765

**Published:** 2022-01-11

**Authors:** Aric Rankin, Andrea Baumann, Bernice Downey, Ruta Valaitis, Amy Montour, Pat Mandy

**Affiliations:** 3710 McMaster University, Hamilton, Canada

**Keywords:** Aboriginal, American Indian/Alaska Native, First Nations, Inuit, Metis, Indigenous, Maori, navigator

## Abstract

**Background:**

Healthcare systems are complex and as a result patients may experience fragmentation of services. Indigenous populations experience increasingly disproportionate health disparities compared to non-Indigenous populations. Patient navigation is known as a patient-centered approach to empower individuals to connect with appropriate services. Literature surrounding the Indigenous Patient Navigator (IPN) remains sparse necessitating this scoping review. Purpose: To map the current state of the role of the IPN internationally within Canada, United States, Australia and New Zealand.

**Methods:**

Estalished methodological framework by Arksey and O’Malley and the PRISMA extension for scoping reviews was used.

**Results:**

A total of 820 articles were reviewed from four databases, yielding sixteen articles.

**Conclusions:**

The absence of published literature surrounding the IPN role in Australia and New Zealand was surprising considering similar histories of colonization. The term navigator was used most often and was typically used when describing lay/peer roles. Professional roles were described using specific role descriptions. Six IPN roles were identified including: (1) social service navigation, (2) wholistic support of Indigenous people, (3) advocacy/building capacity, (4) health assessment, (5) administrative navigation, and (6) outreach. Additionally, barriers and enablers IPNs address are identified. This scoping review will assist to promote and reinforce the IPN role.

## Background

It is estimated that there are between 370 to 500 million Indigenous people from over 90 countries across the world (UNESCO, 2019). Consistently, health disparities among Indigenous populations are increasingly disproportionate in comparison to those of non-Indigenous groups. For example, in Canada, Indigenous populations experience higher morbidity and mortality rates, which has been driven by a two to ten-fold incidence of chronic and infectious diseases (Smylie et al. 2011).

Social determinants of health are the broad range of personal, social, economic and environmental factors that determine individual and population health (Government of Canada 2019). It is the direct impact of these health inequities that burden populations with ill health (Czyzewski 2011). Indigenous peoples have been identified as a vulnerable population^1^ who experience this burden in a much deeper and profound way. Indigenous peoples of Canada are a resilient and vibrant population whose vulnerability stems from the impacts of colonization and assimilative processes such as the devastating and lasting impact of the Indian Residential and Day Schools (CCSDH, 2013). Indigenous-specific social determinants of health identify distal determinants such as inequity produced by social norms, colonial policies and practices that promote and tolerate unfair distribution of resources and profound intergenerational impacts on Indigenous health and wellbeing (Horrill et al. 2019; Reading and Wien 2013). Indigenous health differs from that of the mainstream (non-Indigenous) population. This difference relates to how health and wellness is conceptualized as the balance and inter-relationships of the physical, mental, emotional, and spiritual aspects of a being and not exclusively as an individual and disease process (First Nations Health Authority [FNHA], 2020).

Traditional healing involves the health practices, approaches, knowledge, and beliefs while using ceremonies; plants, animals, or mineral-based medicines; energetic therapies; or physical/hands on techniques (FNHA, 2020). Many Indigenous practitioners including shamans, medicine people, midwives and healers from around the world continue to practice traditional healing methods to manage their collective health. However, due to accessibility issues, awareness or different beliefs, not all Indigenous people choose to utilize these services. For those who have chosen to access a biomedical Western healthcare system, especially in urban settings, health and social disparities as well as the challenges related to navigating a complex healthcare system presents barriers (Allan and Smylie 2015). While the solution to this problem is complex with deep connections to a colonial history of injustices, including the implementation of assimilative policies, improving access to healthcare services is one strategy to bridge the gap of health inequity among Indigenous peoples (Reading and Wien 2013).

In Western society, the role of patient navigation is broadly known as a person-centered approach to empower individuals and families to establish a connection with appropriate services (Freeman and Chu 2005). The term “patient navigation” was first devised in the 1990s by Dr. Harold Freeman to assist a marginalized population of women who experienced barriers while accessing healthcare services (Freeman and Rodriguez 2011). Since then, patient navigation roles and programs have emerged to assist a variety of populations. However, the majority of published research involving the navigator role with Indigenous populations has focused on improving cancer care (Eschiti et al. 2012, Roland et al. 2017). For example, improving cancer diagnosis to treatment times (Dockery et al. 2018).

Navigation in a celestial sense has been central to Indigenous nations across the planet for hundreds of years (Little Bear 2012). The connectedness of communities to the environment through ceremony and other traditional practices is how Indigenous peoples may perceive navigation. The worldviews of mainstream healthcare programs designed for marginalized populations, navigation and otherwise, are influenced by dominant Western worldviews. Traditional knowledge of Indigenous peoples is not taken seriously because it is usually categorized as superstition or folklore (Little Bear 2012). Consideration regarding the development of programs targeted for Indigenous populations is required to avoid paternalism and promote autonomy and self-determination. These are important considerations when utilizing navigation strategies to link Indigenous peoples to mainsteam (Western) and traditional healing paths.

The role of patient navigators is intended to mitigate the barriers to health and social care (Wells and Nuhaily 2018). Patient navigators may be lay or professional navigators. A lay navigator may be from the local community and use their personal expertise as a “cultural broker and interpreter” to assist members of their own community (Wells and Nuhaily 2018). Alternatively, professional navigators are regulated professionals such as nurses or social workers. They may or may not be members of the Indigenous community; however, they provide expertise specific to their professional scope of practice. There is little agreement on which models of patient navigation are best suited to achieve a particular health outcome (Wells and Nuhaily 2018). It has been noted that populations with complex health and social needs were often guided by navigation models with trained professional navigators; whereas, populations where social determinants of health are identified as a priority were often guided by navigation models with lay navigators (Carter et al. 2018). The authors report that this finding speaks to the complexity of needs for health and social service support required in diverse populations and contexts. The term Indigenous Patient Navigator (IPN) will be used in this paper to recognize the unique and diverse cultural differences of Indigenous peoples globally, understanding that despite these differences Indigenous people experience common issues related to colonization and protection of rights as distinct peoples (UNESCO, 2019). Furthermore, an IPN is defined as an individual who assists an individual and/or family who identify as Indigenous. It is important to understand that not all individuals will identify as Indigenous and have the right to self-determination.

The development of the IPN role in North America has been shown to be an effective bridge between Indigenous peoples and Western biomedical healthcare (Grimes et al. 2017). In Canada, the approach of IPN programs is in keeping with the Truth and Reconciliation Commission's (TRC) Call to Action #19, which appeals to governments who aim to establish measurable goals to identify and close the gap of health and social inequities (TRC, 2015). Internationally, this role aligns with other reconciliation movements such as the Council for Aboriginal Reconciliation Act (1991) in Australia, the Waitangi Tribunal (1975) in New Zealand, and similar policies in the United States. While evidence suggests that IPN roles are effective to improve patient satisfaction (Grimes et al. 2017), the literature surrounding how the IPN role addresses the barriers and enablers experienced by Indigenous peoples remains sparse. Given this lack of evidence surrounding the impact of the IPN role, the significant health and social disparities faced by Indigenous communities around the world, and the movement of countries toward reconciliation with Indigenous populations, exploring and understanding the impact of the IPN role related to how it may improve the health of Indigenous communities is needed. Furthermore, this research will provide further evidence related to the IPN role and how this role can address systemic inequities Indigenous peoples face while accessing biomedical Western health and social services in Canada and abroad.

## Methods

The purpose of this scoping review is to: (1) identify the extent and the nature of research pertaining to the role of the IPN in Canada, the United States, Australia, and New Zealand; (2) examine barriers faced by Indigenous peoples when utilizing Western health services; (3) identify potential gaps in the existing published literature and key research priorities, which will assist to inform IPN role development and practice as well as advance related health policies.

A six-step methodological framework outlined by Arksey and O’Malley (2003) was used. These steps include: (1) identifying a research question, (2) identifying relevant studies, (3) carefully selecting studies, (4) charting the data, and (5) collating, summarizing and reporting the results, as well as an optional sixth step to conduct a consultative exercise with stakeholders. Additionally, the PRISMA extension for scoping reviews by Tricco et al. (2018) was used to clarify and enhance Arksey and O’Malley's methodology. These authors are experts within the area of scoping review methods which are situated within a Western research framework. These steps are expanded upon and described below.

Step one involved identifying a research question. The following research question informed this scoping review: What is known in the existing published literature about the role of the Indigenous Patient Navigator with an emphasis on how this role addresses barriers and enablers to health and social services experienced by Indigenous peoples? Additional sub-questions, which are addressed include: What are the roles or functions of the Indigenous Patient Navigator?, and What are the barriers and enablers that Indigenous peoples face while accessing health and social services within a Western biomedical system?

 This scoping review was completed to map the current state of the IPN role internationally within Canada, United States, Australia and New Zealand. Each of these countries have similar colonial histories resulting in marked health and social disparities among Indigenous populations. The combination of Boolean phrase/keywords used for the search strategy included: Indigenous, First Nations, Métis, Inuit, Inuk, Alaska Natives, American Indian, Native American, Indian, Native Hawaiian, Aboriginal, Torres Strait Islander, Pacific Islander, and Maori; patient, client, person, consumer, community, and reserve; navigator, advocate, community representative, community health worker, care coordinator and community health liaison were also used. An academic health sciences librarian with 20 years experience was consulted to develop a robust search strategy.

Step two, identifying relevant studies, was conducted between July 2019 and August 2019 using the online databases Medline, CINAHL, Web of Science, and iPortal.usask.ca. Articles were included if they were published in a peer-reviewed journal, publicly available in full text, available in the English language, published between 1990 and August 2019, and involved lay or professional navigators working with Indigenous individuals and/or families within a geographic location of Canada, the United States, Australia and New Zealand. Articles were excluded if there was not a clear description of the navigator role or if the role was outside the context of the healthcare system. Articles prior to 1990 were excluded because this was the first time the concept of “patient navigation” was published (Freeman and Rodrigues 2011). Finally, editorials, commentary/opinion articles and other forms of grey literature were excluded because the purpose of this scoping review is to identify all peer-reviewed research published within journals surrounding the role of the IPN.

Step three, the careful selection of studies, began with a literature search which yielded 820 articles (Medline: 517; CINAHL: 25; Web of Science: 277; iPortal.usask.ca: 1). Upon review, 293 articles were excluded because they were duplicates. Of the remaining 527 articles, consensus was reached between the two reviewers for 29 articles. One of these reviewers self-identifes as Indigenous. These articles were read and re-read by one of the initial reviewers who does not self-identify as Indigenous, and thirteen articles were excluded because they did not meet the inclusion criteria upon further examination. Additionally, a review of the reference lists and pertinent journals was completed for these articles. No additional articles were identified. Ultimately, sixteen articles were selected for this scoping review.

 Step four, charting the data, was completed by using the thematic framework outlined by Braun and Clarke (2006). Descriptions of recurrent and relevant themes from each full text article were recorded by one reviewer. First, each article was read and re-read in full. Second, a coding process was developed using standardized data charting forms to identify recurrent themes (Braun and Clarke 2006). Study selection is reported according to PRISMA-Sr guidelines (Tricco et al. 2018). For each of the articles the following was charted; authors, year, location by country, study setting, population of interest, study aim, methodology, navigator title, training requirements, lay or professional designation, barriers and facilitators addressed, and study recomendations.

Step five, collating, summarizing and reporting the results, was completed as the abstracted themes and subthemes developed throughout the iterative thematic process (Braun and Clarke 2006). Each of the these themes and sub-themes were grouped together based on the scoping review questions. The results are reported in a narrative summary of study findings.

Finally, step six involved stakeholder consultation which was completed on three occasions throughout the scoping review process with a supervisory committee of five expert researchers, including three Indigenous scholars who are actively involved within the Indigenous community in the areas of research, healthcare and leadership.

## Results

**Figure 1.**
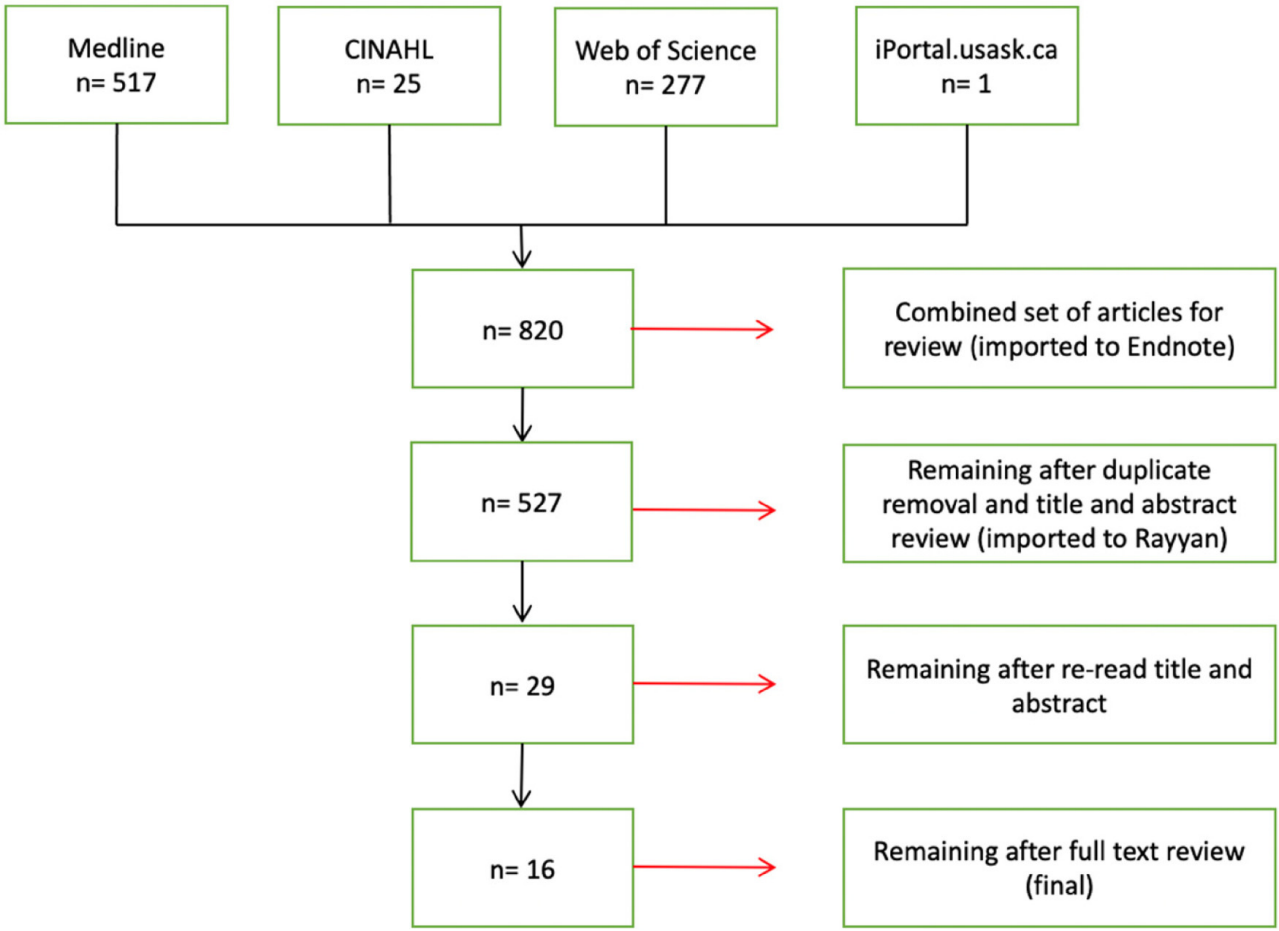
Search Strategy.

The final article selection comprised 16 articles which met eligibility criteria for this scoping review. The majority (n = 12; 75%) of the articles were from the United States, followed by 18.7% (n = 3) from Canada and 6.3% (n = 1) from New Zealand (See Table 1). No articles were identified from Australia. The selected articles ranged in years from 1991 to 2019 with a noticeable gap in publication between 1991 and 2011. A mix between the use of qualitative and quantitative research methods were found including 43.7% (n = 7) qualitative, 37.5% (n = 6) quantitative, and 6.3% (n = 1) used a mixed method convergent design. Additionally, two literature reviews were included. One of the literature reviews focused on cancer care across the continuum and the other focused on two specific cancer care programs (Eschiti et al. 2012), “The Native Sisters Program” and “The Walking Forward Program” (Whop et al. 2012). Both of these literature reviews examined articles from the United States exclusively (Figure 1 PRISMA diagram).

**Table 1.** Specified Article Community.

#	Article	Community
1	Dockery et al. (2018)	American Indian (AI) (not specified)
2	Gampa et al. (2017)	Navajo Navajo Area Indian Health Service
3	Grimes et al. (2017)	AI/AN cancer patients Idaho and Oregon (not specified)
4	Mathu-Muju et al. (2017)	Three communities in Manitoba (not specified)
5	Lavoie et al. (2016)	First Nations Nanaandawe Wigamiq – First Nation Health and Social Secretariat of Manitoba
6	Harjo et al. (2014)	American Indians (AI) (not specified)
7	Kaufmann et al. (2014)	Native Veterans (not specified)
8	Eschiti et al. (2012)	Literature Review
9	Redwood et al. (2012)	Eskimo, Aleut, and American Indian. (not specified)
10	Warren-Mears et al. (2013)	Northwest Tribal communities (not specified)
11	Whop et al. (2012)	Lakota tribes in western South Dakota, AI (not specified)
12	Guadagnolo et al. (2011a)	AI (not specified)
13	Gaudagnolo et al. (2011b)	Oglala Sioux Tribe (Pine Ridge), Cheyenne River Sioux Tribe, Rosebud Sioux Tribe, and the Rapid City American Indian population
14	Lavallee et al. (1991)	Cree
15	Boulton, A.F., Gifford H.H., Potaka-Osborn, M. (2009)	Maori
16	Trevisi, L., Orav, J., E., Atwood, S., Brown, C., Curley, C., King, C., et al. (2019)	Navajo Nation

*9 of 16 articles “not specified”

The following six broad themes were identified: IPN role title, lay or professional IPN roles, IPN clinical setting, IPN role description, barriers and enablers addressed by IPNs, and IPN training (See Table 2). A narrative review of these themes is provided.

**Table 2.** Themes and Sub themes: Barriers and Enablers Addressed by IPNs.

Themes	Sub themes
Barriers	
Systems issues	Distance to healthcare services (n = 7) Underfunding (n = 2) Lack of communication between HCPs (n = 1) Lack of Indigenous specific statistics (n = 1) IPN burnout (n = 3) Lack of awareness of Indigenous benefits (n = 1) Lack of training and support of the IPN role (n = 1) Lack of awareness of the IPN role (n = 1)
Personal issues	Emotional health barriers (n = 1) Mental health barriers (n = 1) Cultural health barriers (n = 4) Physical health barriers (n = 1) Language barriers (n = 2) Mistrust in healthcare providers (HCP) and healthcare system (n = 4) Missing or lost documents (n = 1) Lack of insurance coverage (n = 2) Lack of housing (n = 2) Financial issues (n = 5)
Enablers	
	Cultural familiarity (n = 3) Building trusting relationships (n = 3) Indigenous peoples involvement in IPN role (n = 4) HCP recognition of traditional practices (n = 3) Family supports (n = 1) Health literacy (n = 1) Personal knowledge of the history and resulting trauma in local Indigenous communities (n = 1) Promotion of the IPN role (n = 1)

### IPN role title

The most common title, “patient navigator”, was identified in 50% (n = 8) of the articles. More specifically in the article by Harjo et al. (2014) the role was referred to as “Native Patient Navigator” whereas in the article by Guadagnolo et al. (2011a) the role was entitled “Hospital Based Navigators”. The remaining six articles referred to the role as “Patient Navigator”. The second most common term was “Community Health Representative” which was found in 18.8% (n = 3) of the articles. Closely related to this title, “Community Health Worker” was described by Boulton, Gifford and Potaka-Osborn (2009) and “Community Research Representative” who worked alongside “Hospital Based Navigators” in articles by Guadagnolo et al. (2011a) and Guadagnolo et al. (2011b). Additionally, specific terms were used by Kaufmann et al. (2014) such as “Tribal Veterans Representative” and Mathu-Muju et al. (2017) “Children's Oral Health Initiative Aides”. Finally, Lavoie et al. (2016) used the term “Patient Advocate”.

### Lay or professional IPN roles

Of the sixteen articles, 43.8% (n = 7) included lay IPN roles, whereas 18.8% (n = 3) of the articles included both lay and professional IPN roles. Only 6.3% (n = 1) of the articles included a professional IPN role alone. Additionally, 31.1% (n = 5) of the articles did not identify if the IPN roles were lay or professional.

### Clinical settings of IPN roles

The majority, 75% (n = 12) of the articles identified that the IPNs worked within the cancer care setting. IPNs working in primary care (i.e., primary care provider offices) or community^2^ settings (i.e., home care) were found in 43.7% (n = 7) of the articles, whereas, in 18.7% (n = 3) of studies, the IPN role was embedded within both the primary care/community setting and a tertiary care setting. Moreover, 12.5% (n = 2) of the articles included the IPN role situated within the tertiary setting only. Finally, Redwood et al. (2012) did not indicate the setting the IPN role was based within; however, the research focused on cancer screening, which might indicate a primary care or community setting.

### IPN role description

Six key roles were identified for IPN role descriptions including: social service navigation, wholistic support of Indigenous peoples, advocacy/building capacity, health assessment, administrative navigation, and outreach. These key roles and corresponding activities are described.

Multiple roles of *social service navigation* were described in all sixteen articles. These roles are broken down into seven activities, which include: transportation, housing, financial assistance, arranging referrals and appointments, food acquisition, and arranging childcare. Transportation was identified in 56.2% (n = 9) of the articles. Assistance with housing or accommodations was mentioned in 37.5% (n = 6) of the articles. Financial assistance, including assistance with insurance claims was identified in 43.8% (n = 7) of the articles. Additionally, in 43.8% (n = 7) of the articles, the IPN role assisted with arranging referrals and scheduling appointments. Finally, acquisition of food was mentioned in Lavoie et al. (2016) and arranging childcare was mentioned in Whop et al. (2012). Next, the key role of *wholistic support of Indigenous peoples* was divided into the activities of social, emotional and cultural support. Social support was identified in 12.5% (n = 2) of the articles. Emotional support was found in 25% (n = 4) of the articles. Finally, cultural support^3^ was found in 18.8% (n = 3) of the articles. *Advocacy/building capacity* was another key role of the IPN. Activities included communication with Indigenous peoples and communication with health care providers (HCP). Amongst Indigenous peoples, communication occurs most commonly in the form of individual or group education (n = 9; 56%), followed by resource identification (n = 6; 37%), and appointment reminders (n = 1; 6.3%). Two (12.5%) of the articles found that networking and maintaining relationships with local health agencies was one way to establish communications while IPNs attending medical appointments with Indigenous peoples was also identified as another way to provide communication support (n = 2; 12.5%). The IPN role was described as explaining cultural practices to HCPs and assisting Indigenous peoples to address their questions and concerns in 31% (n = 5) of the articles. Another key role identified for the IPN included *health assessments.* The following activities included; health screening (n = 2; 12.5%), fluoride varnish applications (n = 1; 6.3%), tracking results (n = 5; 31.3%), and providing psychotherapy (n = 1; 6.3%). The key role of *administrative navigation* involved completing paperwork for programs in 25% (n = 4) of the articles, completing insurance forms in 12.5% (n = 2) of the articles and assisting with land issues, such as Treaty rights and self-determination in 6.3% (n = 1) of the articles. The final key role identified for IPN was *outreach* where the IPN was involved in identifying and connecting clients with navigation programs or other services in 25% (n = 4) of the articles.

### IPN training and role requirements

Three (18.8%) of the articles identified educational role requirements such as completion of a certified nursing assistant (CNA) education as well as bilingualism in Navajo and English (Gampa et al. 2017; Trevisi et al. 2019); and 56% (n = 9) of the articles outlined additional organizational training that is provided to IPNs such as cultural competence (Guadagnolo et al. 2011b).

### Barriers and enablers addressed by IPNs

Two major themes related to barriers that Indigenous people face while accessing healthcare services were identified. They include systems issues and personal issues. Sub themes were identified for these major themes (See Table 3).

**Table 3.** IPN Role Description Roles and Activities.

Roles	Activities
Social service navigation	Transportation (n = 9) Housing (n = 5) Financial assistance (n = 7) Arranging referrals and appointments (n = 7) Food acquisition (n = 1) Arranging childcare (n = 1) Interpretation (n = 2)
Wholistic support of Indigenous peoples	Social (n = 2) Emotional (n = 4) Cultural support (n = 3)
Advocacy/Building Capacity	Communication with Indigenous peoples Education (n = 11) Resource identification (n = 6) Appointment reminders (n = 1) Communication with health care providers (HCP) Networking with HCPs (n = 2) Attending medical appointments with peoples (n = 2) Explaining cultural practices to HCPs (n = 5)
Health assessment	Health screening* (n = 2) Fluoride varnish applications* (n = 1) Tracking results* (n = 5) Providing psychotherapy* (n = 1)
Administrative navigation	Completing paperwork for programs (n = 4) Completing insurance forms (n = 2) Assisting with land issues (n = 1)
Outreach	Identifying participants for programs (n = 3)

*navigators had additional education

*Systemic barriers* relate to the complexities of the healthcare system and can be multifactorial. The sub themes for systemic barriers included underfunding (n = 2; 12.5%), lack of communication between care providers (n = 1; 6.3%), lack of Indigenous specific statistics (n = 1; 6.3%), IPN burnout (n = 3; 18.8%), lack of awareness of Indigenous benefits (n = 1; 6.3%), lack of training and support of the IPN role (n = 1; 6.3%), and lack of awareness of the IPN role (n = 1; 6.3%). *Personal barriers* identified for Indigenous peoples receiving healthcare services were divided into sub themes including: emotional health barriers in 6.3% (n = 1), mental health barriers in 6.3% (n = 1), cultural health barriers in 25% (n = 4), and physical health barriers in 6.3% (n = 1) of the articles. Additional barriers included language barriers (n = 2; 12.5%), mistrust in healthcare professionals (HCP) or the healthcare system (HCS) (n = 4; 25%), missing or lost documents (n = 1; 6.3%), lack of insurance coverage (n = 2; 12.5%), lack of housing (n = 2; 12.5%), and financial issues, predominantly related to poverty (n = 5; 31.2%).

Six articles mentioned enablers for Indigenous peoples accessing complex healthcare systems. The major enabler themes included cultural familiarity in 18.7% (n = 3) of the articles, building trusting relationships (n = 3; 18.8%), and Indigenous peoples use and HCP recognition of Indigenous traditional practices (n = 3; 18.8%). Additional enablers identified by Lavoie et al. (2016) included family supports (n = 1; 6.3%), health literacy (n = 1; 6.3%) and finally, Harjo et al. (2014) identified the IPN's personal knowledge of the history and the resulting trauma in the local Indigenous community as an enabler to Indigenous peoples’ health care experience (n = 1; 6.3%).

## Discussion

The purpose of this scoping review was to identify the extent and the nature of research pertaining to the role of the IPN in Canada, the United States, Australia, and New Zealand, examine barriers faced by Indigenous peoples when utilizing Western health services, and identify potential gaps in the existing published literature and key research priorities, which will assist to inform IPN role development and practice as well as advance related health policies.

Given that this scoping review was inclusive of the United States, Canada, Australia and New Zealand, the paucity of literature in Australia and New Zealand was surprising, considering the similar histories of colonization. The exception was a single article from New Zealand (Boulton et al. 2009) where the IPN role included navigating Indigenous governmental policies and frameworks. The initial search strategy identified fifty-two articles from Australia and New Zealand, each which did not meet the inclusion criteria for this review because they did not address patient navigation directly or were not publicly available at the time of this scoping review.

### IPN role title

The term navigator was used most often to describe the title of the IPN role. Other variations included “Native Patient Navigator” and “Hospital Based Navigators”. This was consistent with what other studies have found (Carter et al. 2018). Though a variety of titles were identified, each role focused on addressing barriers faced by Indigenous peoples within the context of a healthcare setting. Having a variety of terms to describe a role creates difficulties for researchers to measure and understand the impact of the role and what it adds to the quality of care (Baumann, 2013). Additionally, role confusion related to range of terms may lead to ineffective utilization of the IPN role including delays for IPN referrals from other members of the healthcare team.

### Lay or professional IPN roles

More than half of the articles included the lay IPN role and only one article included a professional IPN role alone. It is possible that the lower wage of the lay IPN role compared to a professional IPN role is more financially appealing to organizations. According to a literature review published by Eschiti et al. (2012), lay IPNs are less costly and are viewed as more approachable to Indigenous peoples; whereas, professional IPNs may have an easier time understanding and accessing clinical settings in tertiary care settings. Although some articles noted that the lay IPNs self-identified as members of the same Indigenous community they were providing services to, this was not indicated for all the lay or professional IPN roles in the articles in this scoping review. Including lay or professional IPNs who self-identify as Indigenous is an important consideration when designing and providing navigation services. An IPN that does not self-identify as Indigenous may not carry similar worldviews and therefore, have a greater difficulty gaining trust from the Indigenous peoples. In an article by Boulton et al. (2009), being Maori was identified as being “crucial” to IPN effectiveness as members of the healthcare team. Furthermore, given the impacts of trauma and the intergenerational impacts of colonial policies and practices a general distrust has been created with care being provided by non-Indigenous practitioners (Horrill et al. 2019). Stable funding, promotion of the IPN role and recruitment of members of the Indigenous community is required.

Differentiating between lay and professional IPN roles could be determined based upon educational experience. For example, professional IPNs were more likely to have completed college or university programs within the professions of nursing or social work; whereas, lay IPNs had not, but may have completed additional training within their own organization. Additionally, IPN titles that were described more specifically were more likely to involve professional IPN roles, for example, Children's Oral Health Initiative Aides (Mathu-Muju et al. 2017); whereas, IPNs referred to as a “navigator” were more likely to be lay roles. Ultimately, lay IPN and professional IPN roles carry benefits that can be targeted to the healthcare setting and the needs of individual communities navigating a complex healthcare system.

### Clinical setting of IPN roles

The IPN role may be specific to a particular clinical setting or it can be broad in description, formulating the IPN role based upon the needs of the client or community. The majority of the articles reported the IPN role having been implemented in either primary care or community settings or both. Even in the articles where Indigenous peoples attended tertiary settings for treatments the IPN role was involved within the primary care or community setting. It is not surprising given that the need for ongoing IPN support is required once the client has returned home. This observation is likely why more articles including lay IPNs were found within primary care or community settings.

### IPN role description

The IPN role was identified as a way to bridge the gap between Western health care and Indigenous communities in some of the articles (Boulton et al. 2009, Grimes et al. 2017, Gampa et al. 2017, Harjo et al. 2014, Kaufmann et al. 2014). One of the studies outlined that the role was implemented to bridge the gap between translation and the [Indigenous peoples’] fear of the healthcare system and mistrust in health care providers (HCP) (Harjo et al. 2014). Additionally, the IPN role was identified as a patient centered service to overcome barriers (Dockery et al. 2018; Eschiti et al. 2012). Indigenous peoples continue to experience social, economic, cultural, and political inequities which significantly impact their health and wellbeing (Smylie and Firestone 2015). The implications of implementing a role such as the IPN is that it provides an additional voice and support to those they work with to navigate the healthcare system and the systemic racism and structural barriers which continue to be experienced by Indigenous peoples internationally.

The key roles and activities surrounding the work of IPNs included social service navigation, wholistic support of Indigenous peoples, advocacy/capacity building, health assessments, administrative navigation, and outreach.

The role of social service navigation was represented throughout all of the articles. Social service navigation is an appropriate fit for the IPN role as it can assist to address barriers related to social determinants of health including: transportation, housing, financial assistance, arranging referrals and appointments, food acquisition and arranging childcare. The key IPN roles pertaining to the wholistic support of Indigenous peoples and advocacy/capacity building align with the Indigenous social determinants of health. Even though historical colonial policies were ineffective in undermining the unique social value of Indigenous peoples and their desire for self-dermination, there has unfortunately been direct and indirect impacts on health determinants and health status which remain persistent and pervasive (Smylie and Firestone 2015). IPN roles address these distal determinants of health^4^ resulting from inequity produced by social norms and policies and practices that promote and tolerate unfair distributions of resources. For example, the dominant aspects of the IPN role included providing wholistic support such as emotional, social and cultural support to Indigenous people. Interestingly, only three articles identified providing cultural support as one of the IPN activities. The IPN role is situated appropriately within the healthcare system to support Indigenous people and broker relationships with Indigenous Elders and Traditional Knowledge Keepers to provide additional culturally appropriate support.

The health assessment role was reserved for professional IPNs or lay IPNs who have received additional training. Depending on the specific healthcare setting and level of education, the IPN completed health assessments that were within their scope of practice and in accordance with the needs of the Indigenous community. For example, IPNs certified as nurses’ aides to monitor vital signs and measure blood sugars (Gampa et al. 2017) or trained lay IPNs providing fluoride varnish applications (Mathu-Muju et al. 2017). These are additional skills that the IPNs can offer having already developed a trusting relationship. The IPN role of administrative navigation involved activities such as the completion of referrals to programs, insurance forms and other documents. This role was consistent throughout the studies necessitating the need for assistance in this area. Finally, outreach was identified as an IPN role in a quarter of the articles. The activities of the outreach role included engaging Indigenous people and linking them with programs. For example, engaging Indigenous veterans at community events and celebrations to inform them of veteran affairs services (Kaufmann et al. 2014). This is an important role given that the IPN may not be known by other HCPs or Indigenous peoples. Additionally, promotion of the IPN role was an enabler to addressing the various system barriers of Indigenous peoples (Trevisi, et al. 2019).

### IPN training and role requirements

A small proportion of the articles identified IPN role requirements including completion of a certified nursing assistant (CNA) education as well as bilingualism in Navajo and English (Gampa et al. 2017, Trevisi et al. 2019). Another IPN role required education in cultural competence (Guadagnolo et al. 2011b). Finally, Lavallee et al. (1991) required that the IPNs complete a one-year Community Health Representative (CHR) program. It was interesting that only one of the studies included a professional IPN role solely who were trained as Certified Nursing Assistants (Trevisi, et al. 2019), necessitating further research within this area. The majority of the other studies included both lay and professional IPN roles. Additionally, half of the articles outlined additional training to prepare IPNs for their navigation role such as a review of community history and benefits for the IPN program.

### Barriers and enablers addressed by IPNs

Indigenous peoples experience barriers to accessing and receiving care and the IPN role has been identified as a role that can assist clients to navigate the complex healthcare system and address systemic barriers (Eschiti et al. 2012). Two major themes were identified for barriers experienced by Indigenous peoples. They include barriers related to systems issues and barriers related to personal issues. Additionally, structural factors which are imbedded within and systematically produced by the political, historical, social and economic structure of society must also be considered (Reading 2015).

A variety of sub themes were developed in relation to systems issues. The most common sub theme at a systems level involved the distance to healthcare facilities and a lack of available transportation between Indigenous communities and healthcare facilities. This is indicative of the reality of the isolated and rural geographic location of Indigenous communities. This highlights the role of the IPN given their ability to assist with transportation services and link Indigenous peoples with both biomedical and traditional/cultural healthcare services. The report of IPN burnout was mentioned within some of the articles (n = 3) necssitating further research to understand the contributing factors to this issue. Committed resources are required to support IPNs who may experience vicarious trauma, who share similar experiences or who come from the same communities. According to Boulton, Gifford and Potaka-Osborn (2009), IPNs work long hours and often outside their job description. Support for individuals who provide IPN services is integral to the sustainability of the IPN role. The other sub themes identified were specific to each unique context within each study. For example, underfunding of health care system (HCS) was identified and that more research is needed to understand the cost savings of the IPN roles (Eschiti et al. 2012). Other systems issues included a lack of communication between HCPs, lack of Indigenous data, lack of knowledge of Indigenous benefits, a lack of training and support of the IPN role, and a lack of awareness of the IPN role. Further research is required to understand the risks and benefits of providing IPN services and how to best support IPNs by giving them voice and authority to address negative outcomes.

Personal barriers experienced by Indigenous peoples involved the emotional, mental, cultural and physical aspects of personal health. The most common area focused on cultural health barriers. Indigenous health is directly connected to all aspects of the individual including the physical, emotional, mental and spiritual. The IPN explains cultural practices to HCPs and assists Indigenous peoples to have their questions addressed (Grimes et al. 2017; Harjo et al. 2014; Guadagnolo et al. 2011a; Lavallee et al. 1991). Another personal barrier was mistrust with HCPs and the healthcare system. It is likely that this mistrust is closely connected to the inter-generational impact of the colonial histories of oppression and atrocities that have occurred to Indigenous peoples. Having IPNs who self-identify as Indigenous, peoples was identified as a way to assist with creating trust. For IPNs who do not self-identify as Indigenous, additional time and cultural training may be required to develop a trusting relationship between Indigenous peoples, the IPN, HCPs and the healthcare system.

The role of the patient navigator is required to facilitate a culturally safe healthcare experience by mitigating the complexities of the healthcare system and establishing a linkage to the Indigenous community. Given this fact, it should be noted that these roles fall under the umbrella of a predominantly Western system. Navigators work through institutions and their attendant social and power relationships to link individuals to health and social services (Carter, et al. 2018). Boulton et al. (2009), note that the role of the IPN acts as an interface between Western medical science and traditional knowledge and IPNs can be caught between the needs of the community and the expectations of their employer. The IPN role must be driven by the needs of the community and may struggle with the requirements of the larger institutional organization to improve statistics and “compliance” with Western medical interventions. IPN programs must be carefully designed and executed so that a history of colonial injustices are not repeated. These programs should be designed to address specific community needs and with the involvement of Indigenous peoples, particularly Elders and Traditional Knowledge Keepers.

Finally, enablers were identified that positively impacted Indigenous peoples' access to biomedical, Western health and social services. The priority enablers included factors such as IPNs who self-identify as Indigenous peoples, HCP ability to build trusting relationships with Indigenous people, and increasing HCP familiarity with Indigenous culture including awareness of Indigenous traditional practices and knowledge of the history and resulting trauma in Indigenous communities. Additionally, family supports improving health literacy of Indigenous people was also identified as an enabler. Some of these enablers reflect the recommendations by the National Aboriginal Health Organization, whose mandate was to provide health care professionals in Canada with the knowledge and tools to provide culturally safe care while working with Indigenous peoples (Wilson et al. 2013). It is by understanding and building upon these enablers that IPNs can best support Indigenous communities.

Findings from this study can be used to guide future research and in the development of effective IPN role development and IPN program implementation. IPNs play an integral role in connecting Indigenous peoples who choose to access Western or biomedical healthcare to the services they require. This scoping review provides a starting point to work with communities and organizations to reinforce the role of IPNs. Future research is needed that focuses on how the IPN role addresses the individual, system and structural barriers experienced by Indigenous people as they play an integral role to bridge the gap between Western and Traditional health care systems. This scoping review has identified key IPN roles and activities as well as barriers and enablers that Indigenous peoples experience while navigating the healthcare system. Further research is needed to understand and link how the IPN role and activities address the barriers and enablers for Indigenous peoples’ access to biomedical Western healthcare services. Moreover, how the IPN assists to provide cultural support and provide linkages to culturally appropriate wholistic interventions. Finally, a scoping review does not evaluate the quality or weight of evidence; therefore, this review may not provide an objective analysis of the IPN role and its effectiveness and performance necessitating future research within these areas.

## Limitations

A limitation for this scoping review relates to having one primary reviewer for article selection. To mitigate this issue, a validator who self-identified as Indigenous as well as stakeholder consultation with expert researchers and Indigenous stakeholders was completed. The scope of this review was also limited to publicly available full text articles only, which may be a limitation. Finally, because of the unique diversity of Indigenous peoples and Nations around the world, it is challenging to generalize the role of the IPN. Therefore, the reader must be culturally aware of the unique cultural factors related to various Nations implementing and evaluating IPN roles.

## Notes

1. The use of the term vulnerability has a variety of interpretations and a wide field of application in scientific studies (Axelsson & Skold, 2006). The application of this term depends on what population is identified as vulnerable and who defines the term. Identifying and defining a vulnerable population is complex and dependant on contexts (CCSDH, 2013).

2. The term community can be interpreted in a variety of ways. In Canada, a reserve system was imposed on Indigenous peoples by the government. The term reserve and community can be used interchangeably; however, for the purpose of this paper community setting refers to an area outside of the hospital inpatient, acute care setting or hospital clinic setting.

3. The term cultural support refers to the support of an individual’s beliefs, practices, and values. For example access to Elders or tranditional medicines or ceremonies.

4. Reading and Wein (2009), catagorize social determinants of Aboriginal Health into the distal (i.e. historical, political contexts), intermediate (i.e. community infrastructure), and proximal (i.e. health behaviours, physical and social environment). If the determinants of Indigenous health are neglected and ignored we are complicit in the perpetuation of structural inequities that impact the health and wellbeing of Indigenous peoples (Reading, 2015)

## Appendix A

[Table table4-08445621211066765]

**Table table4-08445621211066765:**

Group	Person	Role
Indigenous, First Nations, Métis, Inuit, Inuk, Alaska Natives, American Indian, Native American, Indian, Native Hawaiian, Aboriginal, Torres Strait Islander, Pacific Islander, and Maori	Patient, client, person, consumer, community, and reserve	Navigator, advocate, community representative, community health worker, care coordinator and community health liaison
